# Successful A2 to B Deceased Donor Kidney Transplant after Desensitization for High-Strength Non-HLA Antibody Made Possible by Utilizing a Hepatitis C Positive Donor

**DOI:** 10.1155/2020/3591274

**Published:** 2020-03-13

**Authors:** H. Charli Karpel, Nicole M. Ali, Nikki Lawson, Vasishta S. Tatapudi, Rex Friedlander, Mary Carmelle Philogene, Robert A. Montgomery, Bonnie E. Lonze

**Affiliations:** ^1^Transplant Institute, NYU Langone Health, New York, NY, USA; ^2^The Rogosin Institute, New York, NY, USA; ^3^American Red Cross, Penn Jersey Region, Philadelphia, PA, USA

## Abstract

Desensitization using plasma exchange can remove harmful antibodies prior to transplantation and mitigate risks for hyperacute and severe early acute antibody-mediated rejection. Traditionally, the use of plasma exchange requires a living donor so that the timing of treatments relative to transplant can be planned. Non-HLA antibody is increasingly recognized as capable of causing antibody-mediated renal allograft rejection and has been associated with decreased graft longevity. Our patient had high-strength non-HLA antibody deemed prohibitive to transplantation without desensitization, but no living donors. As the patient was eligible to receive an A2 ABO blood group organ and was willing to accept a hepatitis C positive donor kidney, this afforded a high probability of receiving an offer within a short enough time frame to attempt empiric desensitization in anticipation of a deceased donor transplant. Fifteen plasma exchange treatments were performed before the patient received an organ offer, and the patient was successfully transplanted. Hepatitis C infection was treated posttransplant. No episodes of rejection were observed. At one-year posttransplant, the patient maintains good graft function. In this case, willingness to consider nontraditional donor organs enabled us to mimic living donor desensitization using a deceased donor.

## 1. Introduction

Antibody-mediated rejection remains a major risk factor for graft dysfunction and premature graft failure after kidney transplantation [1, 2]. In the event of humoral rejection, antibodies directed against the donor's human leukocyte antigens (HLA-DSA) are most often sought after to blame. However, multiple independent investigators have reported observations that non-HLA antibodies share in this ability to cause both antibody-mediated injury and antibody-mediated rejection in kidney transplants [3–9]. Anti-endothelial cell antibody (AECA) and antibody directed against the angiotensin II type 1 receptor (AT1R) are among the best characterized non-HLA antibodies [10]. A mounting body of evidence points to the association of non-HLA antibody with risk for rejection and overall poorer long-term outcomes posttransplant [5, 6, 8, 11–19].

Transplantation in the presence of potentially harmful antibodies can be performed under two circumstances: either when the antibody can be removed through desensitization prior to the transplantation, or when an antibody is present at a low enough level that transplant can be safely performed followed by early postoperative desensitization. Plasma exchange is the primary method of desensitization which has been successful at reducing high-strength antibodies prior to incompatible transplantation [20–22]. However, since the effect of plasma exchange is not durable, and antibodies can rebound once treatments are halted, this strategy generally requires a living donor so that the timing of plasma exchange treatments relative to the transplant can be precisely planned. Patients who are broadly sensitized and do not have a living donor are especially disadvantaged. One potential approach might be to initiate empiric plasma exchange for waitlisted patients, but because of the inherent unpredictability of deceased donor organ offers, this strategy is not practically feasible. If one could reasonably predict that an organ offer would be received within a short time frame (a few weeks), it may be possible to start plasma exchange and continue until an offer was received.

One available strategy to increase a recipient's chance of receiving a deceased donor organ offer quickly is willingness to accept an organ from a donor infected with hepatitis C. The advent of direct acting antiviral agents (DAAs) which can cure hepatitis C infection [23, 24] has led to the emerging practice of transplanting hepatitis C infected kidneys into recipients who are hepatitis C naïve and treating with DAAs to cure the infection posttransplant. This practice was borne out of the observation that high-quality hepatitis C positive donor kidneys were being discarded purely due to lack of recipients with hepatitis C infection [25, 26]. Single-center series of hepatitis C positive into negative kidney transplants have been reported with excellent outcomes and, importantly, drastically shorter waiting times to transplant when compared to recipients who waited for a hepatitis C negative offer [25, 27–33].

We present here the case of a patient with AECA, high-strength AT1R antibodies, as well as repeated positive complement dependent cytotoxicity (CDC) crossmatches with all prior deceased donor offers. Given the strength of the AT1R antibody and the potential presence of additional non-HLA antibodies, the patient would have been at increased risk for early acute rejection posttransplant. The patient was willing to accept a hepatitis C positive donor kidney which increased the likelihood of receiving an organ offer quickly. Therefore, we initiated desensitization with plasma exchange to remove the non-HLA antibody. Within weeks of starting desensitization, the patient received a deceased donor kidney transplant from a hepatitis C positive, blood type A2 donor. In this case, a combination of existing and emerging strategies resulted in the successful transplant of a patient who otherwise might have had little hope for an alternative to lifelong dialysis.

## 2. Materials and Methods

### 2.1. Informed Consent

The risks, benefits, and likelihood of success with desensitization were discussed with the patient before initiating plasma exchange, and written informed consent to proceed with plasma exchange and all associated infusions was obtained. With regard to listing patients as eligible to receive hepatitis C positive donor offers, our center has adopted a stepwise approach. First, candidates are extensively counseled regarding the risks and potential benefits of considering organ offers from hepatitis C infected donors. The patients are explicitly told that the expectation is that they will develop hepatitis C infection following transplant. The course of treatment is described, and an assessment is made as to the patient's likelihood of maintaining impeccable compliance with the treatment regimen. If the patient agrees to consider hepatitis C organ offers after this counseling, then the patient provides written authorization expressing willingness to be listed as eligible for hepatitis C donor offers. Once the patient has signed the agreement of willingness to be listed, a check of insurance benefit is performed to ensure there will be coverage for one of the pangenotypic DAAs. Only one insurance benefit is ensured which is the patient's eligibility on the United Network for Organ Sharing (UNOS) waitlist and is changed to reflect that hepatitis C positive donors will be accepted only after this insurance benefit is verified. Importantly, all patient counseling occurs at a specific outpatient consultation visit, so that an informed decision can be made by the patient outside of the context of any pending organ offers. When an organ offer is received, the patients are notified by telephone and are informed at that time of the donor's hepatitis C positive status. If the patient accepts the offer, then one additional specific conversation regarding the risks and benefits of receiving a hepatitis C positive donor kidney occurs between the attending surgeon and the patient, and written informed consent to proceed with kidney transplantation from a hepatitis C positive donor is obtained.

The patient reported here signed a written authorization to be listed as eligible for hepatitis C positive donor organ offers 5 weeks prior to being listed as eligible. In addition, this patient provided written informed consent accepting the hepatitis C positive organ offer on the day of transplant.

### 2.2. Desensitization and Immunosuppression

Desensitization was accomplished by performing serial 1.5 plasma volume exchanges with albumin and/or fresh frozen plasma (FFP) replacement as described elsewhere [22]. The initial frequency was thrice weekly until the AT1R antibody levels decreased. Thereafter, the frequency of plasma exchange was decreased to twice weekly until the patient received the organ offer. At the completion of each plasma exchange treatment, low-dose IVIG (100 mg/kg, Gamunex-C®, Grifols, Research Triangle Park, NC) was administered. Tacrolimus (adjusted for tough level 5-8 mg/mL) and mycophenolate mofetil (2000 mg total daily dose) were initiated with the first plasma exchange treatment. At the time of transplant, induction immunosuppression consisted of antithymocyte globulin (4.5 mg/kg total dose, Thymoglobulin®, Sanofi US, Bridgewater Township, NJ) and pulse corticosteroids. Maintenance immunosuppression consisted of tacrolimus (adjusted to trough level 8-12 ng/mL), mycophenolate mofetil (2000 mg total daily dose), and prednisone (30 mg daily initially tapered to 5 mg daily over the first month postoperatively).

### 2.3. Hepatitis C Virus (HCV) Surveillance and Treatment

Donor blood received with the deceased donor organ was sent for hepatitis C genotyping on the day of transplant. Surveillance screening for recipient hepatitis C viremia by quantitative polymerase chain reaction (PCR) was performed on the day of admission for transplant to confirm the baseline negative. Posttransplant surveillance testing was initiated within the first 7 days of transplant and was continued weekly while on DAA treatment. Treatment was with glecaprevir/pibrentasvir (300 mg/120 mg daily; Mavyret®, Abbvie, Chicago, IL) daily for 12 weeks. At the completion of the 12-week course, surveillance hepatitis C PCR screening was performed weekly for an additional 4 weeks. Thereafter, screening was repeated at 6- and 12-month posttransplant.

### 2.4. Endothelial Cell Crossmatching and AT1R Antibody Testing

Testing for the AT1R antibody was performed using quantitative ELISA (One Lambda, Thermo Fisher, West Hills, CA) as described elsewhere [14]. Endothelial crossmatch assays for the detection of AECA were performed using surrogate blood according to protocols previously reported [19].

## 3. Results

### 3.1. Case Report

The patient is a 46-year-old female with blood type B and end-stage renal disease (ESRD) secondary to lupus nephritis. She was diagnosed with lupus at age 24, treated with prednisone and mycophenolate mofetil. At 39, a kidney biopsy indicated lupus nephritis class III/IV with crescents, and she progressed hemodialysis dependence within 3 years. Additional medical history was notable for mild mitral regurgitation, asthma, and hypertension. No other associated comorbid conditions such as thrombophilia were noted. She had not had any pregnancies nor any blood transfusions. The patient also had no prior exposure to hepatitis C.

She was evaluated at our center and found to be an acceptable candidate for a kidney transplant. As part of the evaluation for transplantation, anti-A2 titers were measured and were less than 1 : 16 (our center's accepted threshold for A2 eligibility), and she was listed as eligible for A2 offers. Over the subsequent months from her listing, she received multiple deceased donor offers from both A2 and B blood type donors. With all offers, she had repeatedly positive B and T cell CDC positive crossmatches despite a panel reactive antibody (PRA) of 0% and no detectable HLA antibody. She was found to have high-strength antibody directed against the angiotensin II type 1 receptor (AT1R Ab > 40 units/mL). Endothelial cell crossmatches were also repeatedly positive with multiple surrogate blood donors. Given the presence of these non-HLA antibodies, we had concern about the risks of attempting transplantation without desensitization prior to transplantation. The patient had no living donors but was A2 eligible, and, most importantly, was willing to accept hepatitis C positive organ offers. Since we were in a rather unusual position of expecting that an offer was likely to present to her quickly, we proposed starting desensitization with plasmapheresis, with the plan that once the non-HLA antibody burden was reduced, and crossmatches with surrogate blood were no longer positive, we would list the patient as eligible for both hepatitis C positive and A2 offers and continue desensitization up until the transplant. Expecting that an offer would come quickly, we could effectively mimic our living donor desensitization protocol [22]. The patient agreed to trial this approach.

Plasma exchange treatments were initiated at a frequency of three times per week. After 5 treatments, the AT1R antibody decreased to a weakly positive level (AT1R Ab 11 units/mL). Endothelial cell crossmatches were performed intermittently, rather than after each plasma exchange treatment. After the 9^th^ plasma exchange treatment, an endothelial cell crossmatch became negative against a surrogate with whom previous tests were repeatedly positive. After these 9 treatments (3 weeks), we reduced the frequency of plasma exchange treatments to twice weekly. After completing 4 weeks of desensitization treatments, the patient was listed as eligible for hepatitis C positive donor organ offers. Within 10 days of listing, an organ became available from a 41-year-old brain dead donor with blood type A2 and both nucleic acid testing (NAT) and antibody testing positive for hepatitis C. The terminal creatinine was 0.6 mg/dL, and the kidney donor profile index (KDPI) was 54. The patient was willing to accept this organ offer and was admitted to the hospital. One immediately pretransplant plasma exchange treatment was performed with FFP replacement. A total of 16 plasma exchange treatments had been performed from the beginning of desensitization to the time of transplant. An endothelial crossmatch against surrogate blood, against which prior crossmatches had been positive, was negative with the most recent serum available prior to transplant. CDC crossmatch with donor blood at the time of transplant was negative. The anti-A2 titer at transplant was 1. The patient underwent an uneventful kidney transplant operation. The final cold ischemia time was 17.9 hours. The kidney began to produce urine, and the recipient's creatinine began to drop immediately. Posttransplant hemodialysis was not required. Three additional plasma exchange treatments were performed on postoperative days 2, 5, and 8.

On postoperative day 3, the patient had detectable hepatitis C viremia. The donor's blood testing revealed a hepatitis C virus with genotype of 1a. Glecaprevir/pibrentasvir was initiated on postoperative day 9 and continued daily for 12 weeks. A peak viral load of 251,000 IU/mL was measured on postoperative day 7. The virus became undetectable by postoperative day 22, which was 13 days after the glecaprevir/pibrentasvir was initiated. The patient has continued to have no detectable hepatitis C viremia since that time.

**Figure 1:**
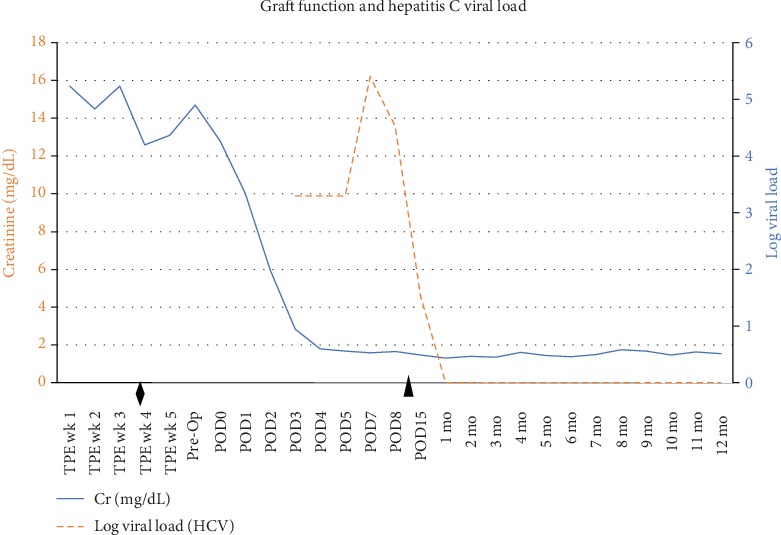
Serum creatinine and hepatitis C viral load before and after transplantation with a hepatitis C positive donor. The patient's serum creatinine normalized without hemodialysis posttransplant and remains 1.6 mg/dL 12-month posttransplant. Hepatitis C viremia was detected on postoperative day 3 with glecaprevir/pibrentasvir treatment initiated on postoperative day 9. Hepatitis C viremia was undetectable by 1-month posttransplant and has remained undetectable at 12-month posttransplant. TPE: therapeutic plasma exchange; POD: postoperative day. ^⧫^Activated for hepatitis C positive offers. ^▲^Initiation of glecaprevir/pibrentasvir.

At 1-month posttransplant, the patient's serum creatinine was 1.32 mg/dL. A kidney biopsy was performed at 4-month posttransplant in the setting of a transient increase in creatinine to 1.8 mg/dL. The biopsy demonstrated no evidence of antibody-mediated rejection and no evidence of cellular rejection. Currently at 12-month posttransplant, the serum creatinine is 1.6 mg/dL (Figure 1).

## 4. Discussion

We report the case of a patient who was desensitized to remove high-strength non-HLA antibody and underwent a successful deceased donor kidney transplant. Non-HLA antibodies are increasingly being recognized as entities that can cause antibody-mediated rejection and lead to accelerated graft loss [14, 17]. In this patient, CDC crossmatches with numerous prior deceased donor offers were repeatedly positive. Whether these lymphocyte-based tests were positive because of the non-HLA antibody is not known but is theoretically possible given that AT1R has been shown to be expressed on lymphocytes [34, 35]. Indeed, hyperacute rejections have been attributed to AECA [19]. In our patient's case, CDC positive crossmatches together with high-strength non-HLA antibody raised concern for the risk of hyperacute rejection without desensitization; however, no living donor was available. Patients in our region (UNOS Region 9) endure among the longest waiting times in the country for a kidney transplant [36]. Here, this nonsensitized patient would have expected to wait potentially years for a standard organ offer. However, because the patient was willing to consider less traditional types of organ offers, we were able to anticipate that an offer would be received relatively quickly. This, in turn, enabled the initiation of empiric desensitization with plasma exchange while an offer was awaited.

In this patient's case, the short wait time from active listing to receiving a transplant (10 days) was enabled by two factors: A2 donor eligibility and hepatitis C positive donor eligibility. The patient's blood type was B, and her anti-A2 titer was within a range deemed acceptable at our center for receipt of a blood type A2 donor kidney. Blood type B recipients have traditionally been the most disadvantaged as they await deceased donor organs. In an effort to offset some of that disadvantage, the 2014 kidney allocation system (KAS) changes enabled eligible blood type B recipients to receive match run priority for A2 deceased donor kidneys [37]. Our center's experience [38], as well as assessments of national organ utilization trends, indicates that these allocation changes have had the desired effect of offering blood type B recipients access to an additional pool of potential deceased donor organs [39].

Ultimately, however, it was the patient's willingness to risk acquiring and acute hepatitis C infection that afforded her the most significant waiting time advantage in this instance. The practice of utilizing hepatitis C infected organs for transplantation into hepatitis C negative recipients has been made feasible by the availability of DAAs which rapidly and effectively eliminate donor-derived hepatitis C viremia posttransplant. The use of hepatitis C positive donor organs for noninfected recipients has broadened since two landmark proof-of-concept studies demonstrated the safety and efficacy of this approach [25, 26]. While these early studies were constructed as formal clinical trials, the favorable risk to benefit balance has led some centers to advocate for offering this option, with proper counseling and consent, as part of the standard of care [29, 30]. As this practice continues to gain traction across the country, the current waiting time advantage may dissipate. But for this case, the option to access this underutilized donor organ pool likely made the difference between a successful transplant and remaining indefinitely on dialysis.
